# A Dirichlet-Multinomial Bayes Classifier for Disease Diagnosis with Microbial Compositions

**DOI:** 10.1128/mSphereDirect.00536-17

**Published:** 2017-12-13

**Authors:** Xiang Gao, Huaiying Lin, Qunfeng Dong

**Affiliations:** a Department of Public Health Sciences, Health Sciences Division, Loyola University Chicago, Maywood, Illinois, USA; b Center for Biomedical Informatics, Health Sciences Division, Loyola University Chicago, Maywood, Illinois, USA; c Bioinformatics Program, Loyola University Chicago, Lake Shore Campus, Chicago, Illinois, USA; d Department of Computer Science, Loyola University Chicago, Water Tower Campus, Chicago, Illinois, USA; University of Wisconsin—Madison; Hanyang University; Simon Fraser University

**Keywords:** Bayes classifier, Dirichlet-multinomial distribution, disease diagnosis, microbiome

## Abstract

Dysbiosis of microbial communities is associated with various human diseases, raising the possibility of using microbial compositions as biomarkers for disease diagnosis. We have developed a Bayes classifier by modeling microbial compositions with Dirichlet-multinomial distributions, which are widely used to model multicategorical count data with extra variation. The parameters of the Dirichlet-multinomial distributions are estimated from training microbiome data sets based on maximum likelihood. The posterior probability of a microbiome sample belonging to a disease or healthy category is calculated based on Bayes’ theorem, using the likelihood values computed from the estimated Dirichlet-multinomial distribution, as well as a prior probability estimated from the training microbiome data set or previously published information on disease prevalence. When tested on real-world microbiome data sets, our method, called DMBC (for Dirichlet-multinomial Bayes classifier), shows better classification accuracy than the only existing Bayesian microbiome classifier based on a Dirichlet-multinomial mixture model and the popular random forest method. The advantage of DMBC is its built-in automatic feature selection, capable of identifying a subset of microbial taxa with the best classification accuracy between different classes of samples based on cross-validation. This unique ability enables DMBC to maintain and even improve its accuracy at modeling species-level taxa. The R package for DMBC is freely available at https://github.com/qunfengdong/DMBC.

**IMPORTANCE** By incorporating prior information on disease prevalence, Bayes classifiers have the potential to estimate disease probability better than other common machine-learning methods. Thus, it is important to develop Bayes classifiers specifically tailored for microbiome data. Our method shows higher classification accuracy than the only existing Bayesian classifier and the popular random forest method, and thus provides an alternative option for using microbial compositions for disease diagnosis.

## Abstract (precis)

By incorporating prior information on disease prevalence, Bayes classifiers have the potential to estimate disease probability better than other common machine-learning methods. Thus, it is important to develop Bayes classifiers specifically tailored for microbiome data. Our method shows higher classification accuracy than the only existing Bayesian classifier and the popular random forest method, and thus provides an alternative option for using microbial compositions for disease diagnosis.

## INTRODUCTION

Various human diseases are associated with dysbiosis in microbial communities, including autoimmune diseases, inflammatory bowel diseases, and obesity (1). Associations between the microbiome and human health and disease raise the possibility of using microbial community compositions as biomarkers for disease diagnosis (2). In principle, standard classification approaches can be used for this purpose, including regression-based methods, support vector machines, random forests, and so on. However, one major limitation of these methods is that they cannot incorporate the prior probability of the disease in the diagnosis (e.g., prevalence of the disease in the general population). Ignoring the prior probability of the disease may often lead to an incorrect medical diagnosis (3). Therefore, Bayes classifiers are particularly relevant for medical diagnosis, since they can incorporate prior disease information for classification and prediction.

The first Bayes classifier for microbiome classification was a multinomial naive Bayes classifier reported by Knights et al. (4). However, the authors of that study did not release any software for public use. Instead, they noted that it is important to develop further novel approaches that leverage natural structures inherent in the microbial community data. This is critical because error rates for Bayes classifiers are considered to be irreducible only if the underlying likelihood models are accurate for the data (5). The multinomial model alone, however, is insufficient to account for the overdispersion in multicategorical count data, whereas the Dirichlet-multinomial (DM) distribution can and is widely used in modeling data sets with extra variation. It has been shown that the DM distribution can effectively model the abundance of taxa in microbiome sequence data (6–10).

In the seminal work published by Holmes et al. (11), the authors developed a Dirichlet-multinomial mixture (DMM) model, in which the parameters for multiple DM distributions are estimated from input data. The implementation of the DMM model resulted in an R package *DirichletMultinomial* (12), which allows users to apply DMM as a Bayes classifier for microbiome classification. The authors of the DMM method evaluated the accuracy of DMM by using human gut microbiome data to discriminate obese subjects from lean subjects. Their results showed that the performance of the DMM model was comparable to the performance of a random forest method (11). It is important to note that DMM uses all of the microbial taxa in the training data sets to build models to discriminate different classes of microbiome samples. Since the number of microbial taxa in the microbiome samples may be very large, particularly at the species level, there can be many parameters to be estimated for DMM as well. This would subsequently require a large amount of training data sets for reliable parameter estimation, which is an unrealistic requirement since human microbiome research is often limited by relatively small sample size. In addition, DMM does not explicitly identify which specific taxa are important for classification. If a subset of taxa that are directly relevant for classification can be identified, such information may be very useful to provide insights for researchers to understand the biological differences between healthy and diseased microbiomes.

In this study, we show that DM distributions combined with automatic feature selection can achieve higher classification accuracy than the DMM method when tested with real-world microbiome data sets.

## RESULTS AND DISCUSSION

As mentioned above, our goal is to develop an improved Bayes classifier, as only a Bayesian classifier allows the incorporation of prior knowledge of disease prevalence into final disease diagnosis. The only existing Bayes classifier specific for microbiome data sets is the DMM program. Therefore, we focused our comparison on DMBC and DMM instead of evaluating every existing machine-learning method. Nonetheless, we have also included a random forest comparison in our study, as random forests have shown better classification accuracies for microbiome data than other popular classification methods, such as support vector machines, elastic nets, and multinomial naive Bayes (9). In our study, we used the *randomForest* package in R (13).

**FIG 1:**
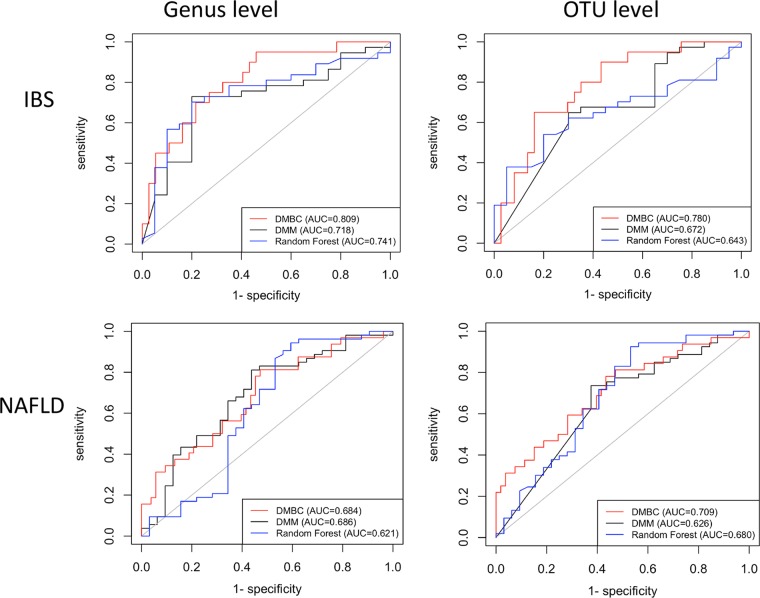
ROC curves for the three classifiers being compared using the IBS and NAFLD data sets at both the genus and OTU levels.

We have used two real-world microbiome data sets for evaluating the classification accuracy of DMBC against other classifiers: the irritable bowel syndrome (IBS) data set and the nonalcoholic fatty liver diseases (NAFLD) data set (see Materials and Methods). Table 1 and Fig. 1 show the corresponding area under the ROC (receiver operating characteristic) curve (AUC) values for the test data sets by DMBC, DMM, and random forest. At the genus level, the classification accuracies of DMBC (i.e., 0.809 for IBS and 0.684 for NAFLD) are higher or comparable to those of DMM (i.e., 0.718 for IBS and 0.686 for NAFLD) and random forest (i.e., 0.741 for IBS and 0.621 for NAFLD). It is important to note that genus-level classification may be very broad; each genus may consist of many species that are not relevant for the particular disease of interest. Therefore, it is important to allow the classifier to also work on species-level data, which may provide higher classification accuracy. However, the number of species-level operational taxonomic units (OTUs), typically based on 3% genetic distance among sequence reads, can be very large from real-world microbiome samples. For example, at the genus level, there are 157 and 120 genera for the IBS and NAFLD data sets, respectively; at the OTU level, there are 6,011 and 4,087 OTUs for the IBS and NAFLD data sets, respectively. The large number of OTUs may be due to the imperfect computational algorithm for denoising and clustering 16S sequence reads, but it is a reality that any microbiome classifier must face. The DMM algorithm considers all the taxa in the data set altogether in its statistical models. This strategy may work for the genus-level taxa, as there are relatively few genus-level taxa, but it may not always work well for a large number of species-level taxa, which poses a challenge for the DMM algorithm to rapidly and accurately estimate the parameters for its statistical models. This challenge is serious when we consider that real-world microbiome sample sizes of the training data sets are typically not very large, i.e., a few hundred at most for the foreseeable future, which is likely insufficient to estimate reliably parameters for a large number of OTUs. Therefore, the advantage of our DMBC’s built-in feature selection step is that DMBC automatically selects a subset of the taxa, which often significantly reduces the number of parameters to be estimated. At the OTU level, the classification accuracies of DMBC (i.e., 0.780 for IBS and 0.709 for NAFLD) are higher or comparable to those of DMM (i.e., 0.718 for IBS and 0.686 for NAFLD) and random forest (i.e., 0.643 for IBS and 0.680 for NAFLD). It is also interesting to note that the classification accuracies of DMM tend to deteriorate at the OTU level when comparing to those at the genus level, while the accuracies of DMBC may be stable or even higher at the OTU level compared to the genus level (Table 1). It is also worth mentioning that the AUC values based on selected taxon features are higher than those based on all the taxa at either the genus or OTU level for both the IBS and NAFLD data sets (data not shown).

**TABLE 1:** Comparison of the classification accuracies between DMBC, DMM, and random forest methods

Test data set^a^	Classification accuracy (AUC)^b^		
	DMBC	DMM	Random forest
IBS at the genus level (157 genera)	0.809	0.718	0.741 (0.005)
IBS at the OTU level (6,011 OTUs)	0.78	0.672	0.643 (0.008)
NAFLD at the genus level (120 genera)	0.684	0.686	0.621 (0.006)
NAFLD at the OTU level (4,287 OTUs)	0.709	0.626	0.680 (0.004)

a For each test data set, the taxonomic level (genus- or species-level OTU) andthe number of features (i.e., the number of genera or OTUs) are indicated.

b The classification accuracies, computed with leave-one-out cross validation, are represented by the AUC values for each classifier. Since the results of the random forest method are affected in its intrinsic random generation of the decision trees, we repeated each random forest classification three times and reported averages with the corresponding standard deviations in parentheses.

In addition to the IBS and NAFLD data sets, we have also attempted to compare DMBC and DMM with an additional published human microbiome data set, the colorectal carcinoma (CC) data set (SRA accession no. SRP000383) consisting of microbiota from 90 carcinoma samples and 95 healthy controls (14). There are 211 genera in the CC data set. For the genus-level CC data set, the AUC value of DMBC is higher than that of DMM: 0.78 for DMBC and 0.73 for DMM. There are 40,007 species-level OTUs in the CC data set. Despite the large number of OTUs, DMBC finished computation in about 3 days and obtained an AUC value of 0.96. However, for the same OTU-level data set, we had to terminate DMM, as it could not finish computation after 3 months, to prevent any further delay of the completion of this study. It is unclear why DMM has trouble with the OTU-level CC data set. We speculate that it is due to the large number of OTUs, which presents a challenge for its optimization algorithm for complicated parameter estimations. In contrast, the automatic feature selection function of our DMBC method significantly reduced the number of OTUs in the DM model from 40,007 OTUs in the original data set to a final subset of 22 signature OTUs with the maximum capability to discriminate diseased and healthy microbiome samples.

Since DMBC simultaneously identifies a subset of taxa that are the most significant in discriminating healthy and diseased microbiome samples, this may potentially provide valuable biological insights on the difference between a healthy and diseased microbiome. For example, the genera *Dialister* and *Bifidobacterium* were identified by DMBC as signature bacteria from the IBS data sets, which is consistent with previous results by Jeffery et al. (15), showing that both genera were significantly different in IBS patients and healthy controls. Similarly, the genera *Alistipes* and *Prevotella* were identified by DMBC as signature bacteria from the NAFLD data sets, which is consistent with the previous results by Jiang et al. (16), showing that both genera were significantly different between NAFLD patients and healthy controls.

We recognize that our current method relies on the maximum likelihood approach in order to estimate parameters for the DM models. Although our method achieves higher accuracy than the available DMM method, we plan to explore the full Bayesian method in the future for parameter estimation to further improve our software, especially when we need to integrate other medical data in addition to the microbiome data for the disease diagnosis.

In summary, we have developed a new Bayes classifier based upon the DM distribution with automatic feature selection, which provides an important option for researchers to use microbial compositions for disease diagnosis. For clinical application, additional work is required to improve the classification accuracy, but DMBC provides an alternative option on further improving Bayes classifiers for microbiome data sets. The DMBC is implemented in R (17), which is freely available at https://github.com/qunfengdong/DMBC under the GNU General Public License.

## MATERIALS AND METHODS

The DM distribution (8, 9, 18) is defined as

(1) $$P(s;N,p)=\frac{N!}{\Pi_{t=1}^{T}\;s_{t}!}\Pi_{t=1}^{T}\;{p_{t}}^{s_{t}}$$

where *T* is the total number of microbial taxa (i.e., the number of categories) identified from the sequencing, *N* is the total number of sequence reads (i.e., *N* independent trials); ***s*** = (*s*_1_, …, *s*_*t*_), representing the number of sequence reads corresponding to each individual category of microbial taxon *t*, satisfying $N=\overset{T}{\underset{t=1}{\sum }}\;s_{t}$; and ***p*** = (*p*_1_,* …*, *p*_*t*_), representing the probabilities that a randomly selected sequence belongs to a microbial taxon *t*, satisfying $\overset{T}{\underset{t=1}{\sum }}\;p_{t}=1$. The parameters for the DM distributions in equation 1 can be estimated from input data by using maximum likelihood approaches (9, 19, 20). We use the R package *dirmult* (19) for estimating the DM parameters from the training data sets. Once the DM parameters are estimated for different categories of samples (e.g., healthy and disease categories), Bayes’ theorem can be applied as follows

(2) Pr(C|M)=Pr(M|C)Pr(C)/Pr(M)

where Pr(*C* | *M*) is the posterior probability that observed microbiome data, *M* (i.e., the observed number of sequence reads for each microbial taxon), belongs to a particular category, *C*, e.g., a disease category; Pr(*M* | *C*) is the likelihood of observing the microbiome data *M* if it was derived from the category *C*; Pr(C) is the prior probability of category *C*, e.g., the prevalence of the disease in the general public based on previous knowledge. The likelihood Pr(*M* | *C*) can be computed once the DM parameters (i.e., *p*) are estimated for the category *C* from the training data sets using equation 1 as described above. Pr(M) is the marginal distribution of the microbiome data *M* to be classified. This can be calculated based on the law of total probability as the summation of the product of likelihoods and prior probabilities of all the categories, i.e., $\sum_{i=1}^{m}\text{Pr}(M\;|\;C_{i})\text{Pr}\left(C_{i}\right)$ for *m* total categories in which the microbiome data set *M* is derived from. In the simplest case, there are only two categories (i.e., *m* = 2): healthy (*C*_1_) and diseased (*C*_2_).

**FIG 2:**
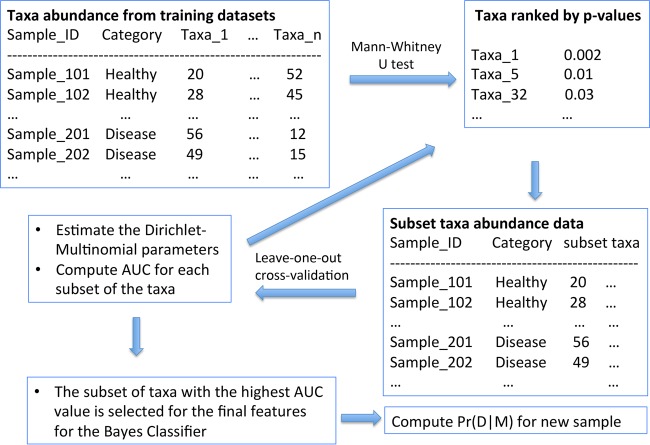
Overview of the DMBC method. A major characteristic of our method is to automatically select a subset of microbial taxa that may achieve the highest classification accuracy (i.e., feature selection). See Materials and Methods for details.

The overview of the DMBC method is illustrated in Fig. 2. Users start by providing a training data set consisting of two categories of data, e.g., disease and healthy microbial compositions. Typically, 16S rRNA gene sequencing is used in microbiome studies for characterizing bacterial community compositions. Bioinformatic software such as mothur (21) and QIIME (22) can be used for either classifying 16S rRNA gene sequence reads at various taxonomic levels or clustering highly similar 16S rRNA gene sequences into species-level operational taxonomic units (OTUs). Our current implementation focuses on binary classification (i.e., disease versus healthy). Each training data set contains the label (e.g., healthy or diseased sample) and taxon abundance (e.g., the number of 16S sequence reads corresponding to each bacterial taxa in each microbiome sample). A common practice in microbiome analysis is to perform subsampling to ensure that the sequencing depth is equal among different samples. However, subsampling is not necessary for DMBC, which works for taxa frequency based on either the original sequencing depth or subsampled sequence data, and classification results are similar (data not shown).

The next step of the DMBC algorithm is to perform the Mann-Whitney U test to compare the abundance of each taxon between the two categories of samples. In a future study, we will explore using parametric-distribution-based regression methods (e.g., negative binomial regression) to replace the Mann-Whitney U test in the DMBC algorithm. The advantage of regression methods includes the potential of taking covariates (e.g., age and gender) into consideration, although the checking of model fit is required and is not always practical to automate for a large number of taxa. On the other hand, the nonparametric Mann-Whitney U test is more robust, since it does not assume any particular distribution for the taxon abundance. The Mann-Whitney U test has also been shown, by the popular LEfSe program (23), to successfully identify microbial taxa as biomarkers whose abundances are significantly different between different categories of microbiome samples. In addition, although parametric models have the potential of producing more accurate *P* values if the models really fit the data, the absolute quantities of the *P* values are not critical for the DMBC algorithm. DMBC simply uses the *P* values to rank the taxa for feature selection instead of for traditional statistical hypothesis testing. Additionally, as described below, the importance of each taxon (i.e., feature selection) will be evaluated using cross-validation; therefore, multiple test correction of the *P* values is not necessary.

The taxa are ranked according to their *P* values from the smallest to the largest. The feature selection is an iterated process as follows. In the first round, the abundance of the taxon with the smallest *P* value is saved, and the abundances of all other taxa are merged together. In other words, the original taxon abundance data set is reduced to a new two-column data set, in which the first column corresponds to the abundance of the taxon with the smallest *P* value and the second column corresponds to the merged abundance of all the other taxa. Then, the leave-one-out cross-validation is performed with this new data set, and its area under the ROC curve (AUC) value is recorded. The rationale behind the first round of the process is to examine how effectively the taxon with the smallest *P* value can be used for the classification purpose. In the next round, the abundances of the two taxa with the smallest *P* value and the second-smallest *P* value are saved individually, and the abundances of all other taxa are merged, i.e., a new three-column data set for obtaining its AUC value. The purpose of the second round of process is to examine classification effectiveness based on the above two selected taxa. This process is iterated until the abundances of all of the taxa whose *P* values are smaller than a threshold (default is 0.5) are saved individually, and AUC values are recorded for each round of the iteration. The set of the individually saved taxa with the highest AUC values is then selected as the most important group of taxa (i.e., signature taxa) for estimating the final parameters of the DM distribution using all the training data sets. The classification of new microbiome samples is obtained by simply calculating the posterior probability using equation 2 based on the final DM distributions.

The following two real-world microbiome data sets, selected on the basis of the availability of the raw sequences and metadata, were used for evaluating the classification accuracy of the three classifiers: (i) the nonalcoholic fatty liver diseases (NAFLD) data set (SRA accession no. SRP041721) consisting of fecal microbiota from 53 NAFLD patients and 32 healthy controls (16) and (ii) the irritable bowel syndrome (IBS) data set (provided by Paul O’Toole via personal communication) consisting of fecal microbiota from 37 IBS patients and 20 healthy controls (15). For testing purposes, we followed the same procedure used in the original DMM publication to estimate the prior probability of each category from the training data sets, i.e., the relative frequency of each category in the entire training data set. It is important to note that users of our DMBC package have the option to specify the prior probabilities based on previous research results, e.g., the disease prevalence information.

The 16S sequences from the two above data sets were processed by the mothur package (version 1.37.4) to remove low-quality and chimeric sequences by following mothur’s standard operating procedures with default parameters (http://www.mothur.org). Species-level OTUs were clustered based on the commonly used 97% similarity cutoff. Genus-level taxonomic classification was performed using the RDP Classifier (version 2.11) (24) with the default cutoff value of 0.8 on the high-quality 16S sequences from each sample.

## References

[1] Pevsner-Fischer M, Tuganbaev T, Meijer M, Zhang S, Zeng Z, Chen M, Elinav E 2016 Role of the microbiome in non-gastrointestinal cancers. World J Clin Oncol 7:200. doi:10.5306/wjco.v7.i2.200. 27081642 PMC4826965

[2] Dietert RR, Silbergeld EK 2015 Biomarkers for the 21st century: listening to the microbiome. Toxicol Sci 144:208–216. doi:10.1093/toxsci/kfv013. 25795652

[3] Banerjee A, Jadhav SL, Bhawalkar JS 2009 Probability, clinical decision making and hypothesis testing. Ind Psychiatry J 18:64–69. doi:10.4103/0972-6748.57864. 21234167 PMC3016704

[4] Knights D, Costello EK, Knight R 2011 Supervised classification of human microbiota. FEMS Microbiol Rev 35:343–359. doi:10.1111/j.1574-6976.2010.00251.x. 21039646

[5] Tumer K, Ghosh J 1996 Estimating the Bayes error rate through classifier combining, p 695–699. *In* Proceedings of the 13th International Conference on Pattern Recognition, vol 2 IEEE, New York, NY.

[6] Knights D, Kuczynski J, Charlson ES, Zaneveld J, Mozer MC, Collman RG, Bushman FD, Knight R, Kelley ST 2011 Bayesian community-wide culture-independent microbial source tracking. Nat Methods 8:761–763. doi:10.1038/nmeth.1650. 21765408 PMC3791591

[7] La Rosa PS, Brooks JP, Deych E, Boone EL, Edwards DJ, Wang Q, Sodergren E, Weinstock G, Shannon WD 2012 Hypothesis testing and power calculations for taxonomic-based human microbiome data. PLoS One 7:e52078. doi:10.1371/journal.pone.0052078. 23284876 PMC3527355

[8] Chen J, Li H 2013 Variable selection for sparse Dirichlet-multinomial regression with an application to microbiome data analysis. Ann Appl Stat 7:418–442. doi:10.1214/12-AOAS592. PMC3846354 24312162

[9] Yu P, Shaw CA 2014 An efficient algorithm for accurate computation of the Dirichlet-multinomial log-likelihood function. Bioinformatics 30:1547–1554. doi:10.1093/bioinformatics/btu079. 24519380 PMC4081639

[10] Chen J, Bittinger K, Charlson ES, Hoffmann C, Lewis J, Wu GD, Collman RG, Bushman FD, Li H 2012 Associating microbiome composition with environmental covariates using generalized UniFrac distances. Bioinformatics 28:2106–2113. doi:10.1093/bioinformatics/bts342. 22711789 PMC3413390

[11] Holmes I, Harris K, Quince C 2012 DirichletMultinomial mixtures: generative models for microbial metagenomics. PLoS One 7:e30126. doi:10.1371/journal.pone.0030126. 22319561 PMC3272020

[12] Morgan M 2014 Dirichlet multinomial: Dirichlet-multinomial mixture model machine learning for microbiome data. R package. R Foundation for Statistical Computing, Vienna, Austria.

[13] Liaw A, Wiener M 2002 Classification and regression by randomForest. R News 2:18–22.

[14] Kostic AD, Gevers D, Pedamallu CS, Michaud M, Duke F, Earl AM, Ojesina AI, Jung J, Bass AJ, Tabernero J, Baselga J, Liu C, Shivdasani RA, Ogino S, Birren BW, Huttenhower C, Garrett WS, Meyerson M 2012 Genomic analysis identifies association of Fusobacterium with colorectal carcinoma. Genome Res 22:292–298. doi:10.1101/gr.126573.111. 22009990 PMC3266036

[15] Jeffery IB, O’Toole PW, Öhman L, Claesson MJ, Deane J, Quigley EM, Simrén M 2012 An irritable bowel syndrome subtype defined by species-specific alterations in faecal microbiota. Gut 61:997–1006. doi:10.1136/gutjnl-2011-301501. 22180058

[16] Jiang W, Wu N, Wang X, Chi Y, Zhang Y, Qiu X, Hu Y, Li J, Liu Y 2015 Dysbiosis gut microbiota associated with inflammation and impaired mucosal immune function in intestine of humans with non-alcoholic fatty liver disease. Sci Rep 5:8096. doi:10.1038/srep08096. 25644696 PMC4314632

[17] R Core Team 2014 R: a language and environment for statistical computing. R Foundation for Statistical Computing, Vienna, Austria.

[18] Mosimann JE 1962 On the compound multinomial distribution, the multivariate β-distribution, and correlations among proportions. Biometrika 49:65–82. doi:10.1093/biomet/49.1-2.65.

[19] Tvedebrink T 2010 Overdispersion in allelic counts and θ-correction in forensic genetics. Theor Popul Biol 78:200–210. doi:10.1016/j.tpb.2010.07.002. 20633572

[20] Sklar M 2014 Fast MLE computation for the Dirichlet multinomial. arXiv arXiv:1405.0099 https://arxiv.org/abs/1405.0099v1.

[21] Schloss PD, Westcott SL, Ryabin T, Hall JR, Hartmann M, Hollister EB, Lesniewski RA, Oakley BB, Parks DH, Robinson CJ, Sahl JW, Stres B, Thallinger GG, Van Horn DJ, Weber CF 2009 Introducing mothur: open-source, platform-independent, community-supported software for describing and comparing microbial communities. Appl Environ Microbiol 75:7537–7541. doi:10.1128/AEM.01541-09. 19801464 PMC2786419

[22] Caporaso JG, Kuczynski J, Stombaugh J, Bittinger K, Bushman FD, Costello EK, Fierer N, Peña AG, Goodrich JK, Gordon JI, Huttley GA, Kelley ST, Knights D, Koenig JE, Ley RE, Lozupone CA, McDonald D, Muegge BD, Pirrung M, Reeder J, Sevinsky JR, Turnbaugh PJ, Walters WA, Widmann J, Yatsunenko T, Zaneveld J, Knight R 2010 QIIME allows analysis of high-throughput community sequencing data. Nat Methods 7:335–336. doi:10.1038/nmeth.f.303. 20383131 PMC3156573

[23] Segata N, Izard J, Waldron L, Gevers D, Miropolsky L, Garrett WS, Huttenhower C 2011 Metagenomic biomarker discovery and explanation. Genome Biol 12:R60. doi:10.1186/gb-2011-12-6-r60. 21702898 PMC3218848

[24] Wang Q, Garrity GM, Tiedje JM, Cole JR 2007 Naive Bayesian classifier for rapid assignment of rRNA sequences into the new bacterial taxonomy. Appl Environ Microbiol 73:5261–5267. doi:10.1128/AEM.00062-07. 17586664 PMC1950982

